# Eggs sampling as an effective tool for identifying the incidence of viruses in honey bees involved in artificial queen rearing

**DOI:** 10.1038/s41598-024-60135-1

**Published:** 2024-04-26

**Authors:** Caio E. C. Domingues, Laura Šimenc, Ivan Toplak, Dirk C. de Graaf, Lina De Smet, Wim Verbeke, Luc Peelman, Leticia S. Ansaloni, Aleš Gregorc

**Affiliations:** 1 Faculty of Agriculture and Life Sciences, University of Maribor, Pivola 10, 2311 Hoče, Slovenia; 2 Institute of Microbiology and Parasitology, Veterinary Faculty, University of Ljubljana, Gerbičeva 60, 1000 Ljubljana, Slovenia; 3 Laboratory of Molecular Entomology and Bee Pathology, Ghent University, Krijgslaan 281 S2, 9000 Ghent, Belgium; 4 Department of Agricultural Economics, Ghent University, Coupure Links 653, 9000 Ghent, Belgium; 5 Laboratory of Animal Genetics, Department of Veterinary and Biosciences, Ghent University, Heidestraat 19, 9820 Merelbeke, Belgium

**Keywords:** Ecology, Molecular biology, Zoology, Ecology, Environmental sciences

## Abstract

The Carniolan honey bee (*Apis mellifera carnica*) plays an essential role in crop pollination, environment diversity, and the production of honey bee products. However, the health of individual honey bees and their colonies is under pressure due to multiple stressors, including viruses as a significant threat to bees. Monitoring various virus infections could be a crucial selection tool during queen rearing. In the present study, samples from all developmental stages (eggs, larvae, pupae, and queens) were screened for the incidence of seven viruses during queen rearing in Slovenia. The screening of a total of 108 samples from five queen breeders was performed by the RT-qPCR assays. The results showed that the highest incidence was observed for black queen cell virus (BQCV), Lake Sinai virus 3 (LSV3), deformed wing virus B (DWV-B), and sacbrood virus (SBV). The highest viral load was detected in queens (6.07 log_10_ copies/queen) and larvae (5.50 log_10_ copies/larva) for BQCV, followed by SBV in larvae (5.47 log_10_ copies/larva). When comparing all the honey bee developmental stages, the eggs exhibited general screening for virus incidence and load in queen mother colonies. The results suggest that analyzing eggs is a good indicator of resilience to virus infection during queen development.

## Introduction

The honey bee (*Apis mellifera*, Linnaeus, 1758) plays a crucial role as a pollinator in agriculture for a wide range of crops and in the natural environment. Nevertheless, they are exposed to multiple stressors^1–3^. Improvements are needed in the data related to honey bee disease appearances and in the experimental data and insights concerning infection modes, pathogen transmission, and persistence^4^. Viruses, however, pose a significant threat to honey bee populations as they can cause death of individual bees or even the collapse of entire colonies, often without any noticeable clinical symptoms^5–7^. Viruses represent a highly diverse group of pathogens that affect honey bees, with over 20 known species infecting both brood and adults of *A. mellifera* worldwide^8^. These viruses represent significant threats to the health and well‐being of honey bees^9^.

Several honey bee viruses have had their genome sequences determined, including the acute bee paralysis virus (ABPV), black queen cell virus (BQCV), chronic bee paralysis virus (CBPV), deformed wing virus (DWV), Israeli acute paralysis virus (IAPV), Kashmir bee virus (KBV), Lake Sinai virus (LSV), sacbrood virus (SBV), and slow bee paralysis virus (SBPV)^10–14^. Viruses can exist or coexist within individual bees or colonies, and while some viruses do not induce clinical symptoms^9,15,16^, they can cause closed infections in honey bees^17^. On the other hand, some viruses exhibit apparent clinical symptoms^9^.

SBV is a significant causative agent of viral diseases in honey bee colonies, characterized by distinctive and well-defined clinical symptoms that affect the brood^18^. Nevertheless, SBV may also be found in adults without apparent signs of infection^19^. CBPV, the first bee virus to be isolated and described, is an example of a contagious disease in adult honey bees, manifesting as chronic paralysis syndrome, ultimately leading to the death of infected bees^20^. The number of reported cases has been increasing in recent decades in England, Wales^21^, the United States of America^22^, and Italy^23^. Furthermore, CBPV can persist asymptomatically in bees^24^. Similarly, ABPV can be found in apparently healthy bees, but it is also associated with the sudden collapse of honey bee colonies due to heavy infestations of varroa^25^. ABPV has been detected in adult bees and varroa in severely varroa-infested colonies^26,27^.

Amidst this backdrop, the most widespread and commonly detected virus in honey bees, DWV, is observed in varroa-infested colonies throughout Europe^28^. Bees may have a shortened lifespan due to deformities that can cause deformed wings during their developmental stages, affecting the colony's dynamics^28–30^. On the other hand, BQCV and SBV are commonly found in adult bees and impact queen prepupae or pupae, especially during the spring and early summer. BQCV has been identified as a common cause of queen larval death in Australia, detected in deceased honey bee queen larvae and pupae^31^. LSV was associated with the weakening of honey bee colonies in the United States of America. Viral infections are a significant concern as they can also cause damage during the developmental stages of honey bees, including eggs, larvae, and pupae, and this in adult workers, drones, or queens^4,32^.

The Carniolan honey bee, native to Slovenia, is a subspecies of *A. mellifera* that is reared to ensure high quality and purity^33^. Many studies of screening for viruses in Carniolan queens have been conducted, with a focus on the presence of viral strains such as ABPV, BQCV, SBV, DWV, and CBPV^33–36^. The most prevalent virus detected in Slovenian queen-rearing operations has been BQCV. According to Gregorc and Bakonyi^33^, the detection rate for BQCV shows a wide range, varying from 0 to 100%. Previous studies have revealed the presence of ABPV, BQCV, and DWV in both workers and queens during queen-rearing procedures. A high incidence of BQCV, 100%, was observed in workers in mating nuclei, while the incidence of the same virus in queens was 50%^35^.

In honey bee colonies, viruses can be transmitted through horizontal and vertical pathways, sometimes coinciding^4^. Horizontal transmission is characterized by interactions among individuals within the same generation, such as the exchange of food by trophallaxis, as well as air and food contamination and also from intermediate biological hosts, such as varroa mites^4^. On the other hand, vertical transmission is characterized between generations, from mother to brood^37^. de Graaf et al.^38^ pointed out for the first time that a novel trait, ‘Suppressed in ovo virus infection’ (SOV) in drone eggs, reflects the potential heritability of virus infection control by honey bees. Other studies have also confirmed the efficiency of the SOV trait^39,40^.

Although there are still many gaps in our understanding of bee virus appearance during the developmental stages of queens, it is crucial to conduct studies that focus on elucidating the occurrence of these viruses in queen rearing practices. The knowledge that such studies may yield has substantial implications for the quality of reared queens and improves our understanding of epizootiology in honey bee queens during development. Our research consisted of conducting experiments under actual queen-rearing conditions in apiaries, with multiple sampling points, to determine the incidence of viruses in various developmental stages, including eggs, larvae, pupae, and young virgin queens. The present study elucidates the current situation and epizootiology of seven bee viruses (ABPV, BQCV, CBPV, DWV-A, DWV-B, LSV3, and SBV) during the queen’s development. We examined queens’ developmental stages collected from several bee colonies across Slovenia for studying selected honey bee viruses in queen-rearing apiaries.

## Results

The results demonstrated the calculation of virus incidence in eggs, larvae, pupae, and newly emerged queens. All samples of brood and queens proceeded were alive, without clinical symptoms of any morphological or other changes caused by diseases.

### Virus incidence in pooled queen's developmental stages

**Figure 1:**
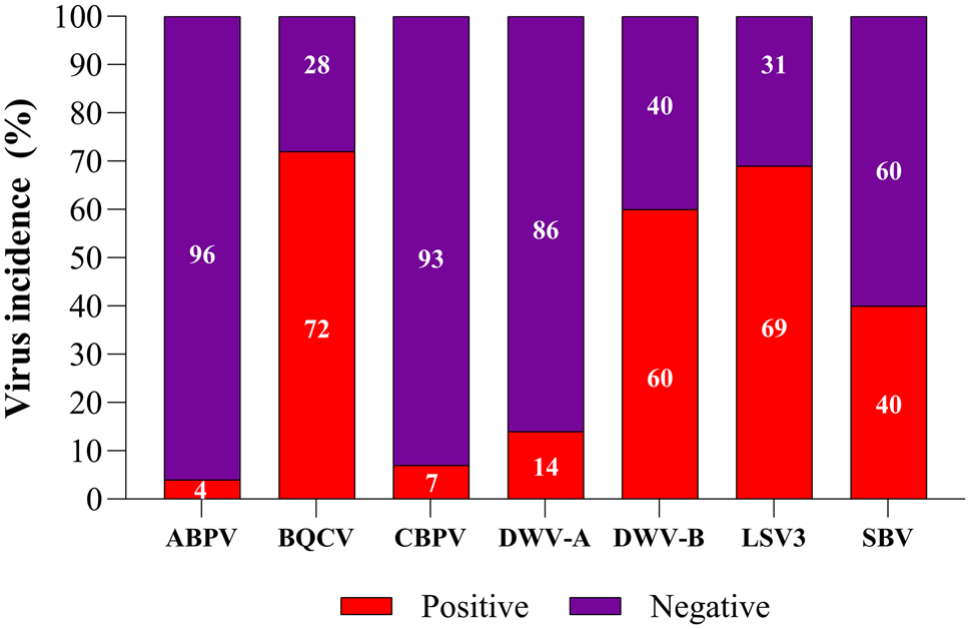
The detected virus incidence of ABPV, BQCV, CBPV, DWV-A, DWV-B, LSV3, and SBV in pooled queen's developmental stages (eggs, larvae, pupae, and queens) collected from five queen breeders. A total of 108 samples from 27 colonies were tested and analyzed. 95% confidence intervals for virus incidence: ABPV (0.011–0.069), BQCV (0.626–0.814), CBPV (0.025–0.129), DWV-A (0.079–0.201), DWV-B (0.51–0.69), LSV3 (0.614–0.766), and SBV (0.283–0.517).

Considering tested pooled samples of developmental stages (eggs, larvae, pupae, and queens) from queen mother colonies from five queen breeders, it was possible to get an overview of the viruses' incidence (ABPV, BQCV, CBPV, DWV-A, DWV-B, LSV3, and SBV) in queen rearing. An incidence of over 50% was observed for three viruses (BQCV, DWV-B, and LSV3), while four viruses (ABPV, CBPV, DWV-A, and SBV) appeared in less than 50% of tested samples of queen developmental stages as shown in Fig. 1. The results of Spearman’s correlation analysis of viruses detected in pooled samples can be accessed in the supplementary information (see Supplementary Table S1).

### Virus incidence and viral loads detected in different stages of development

**Figure 2:**
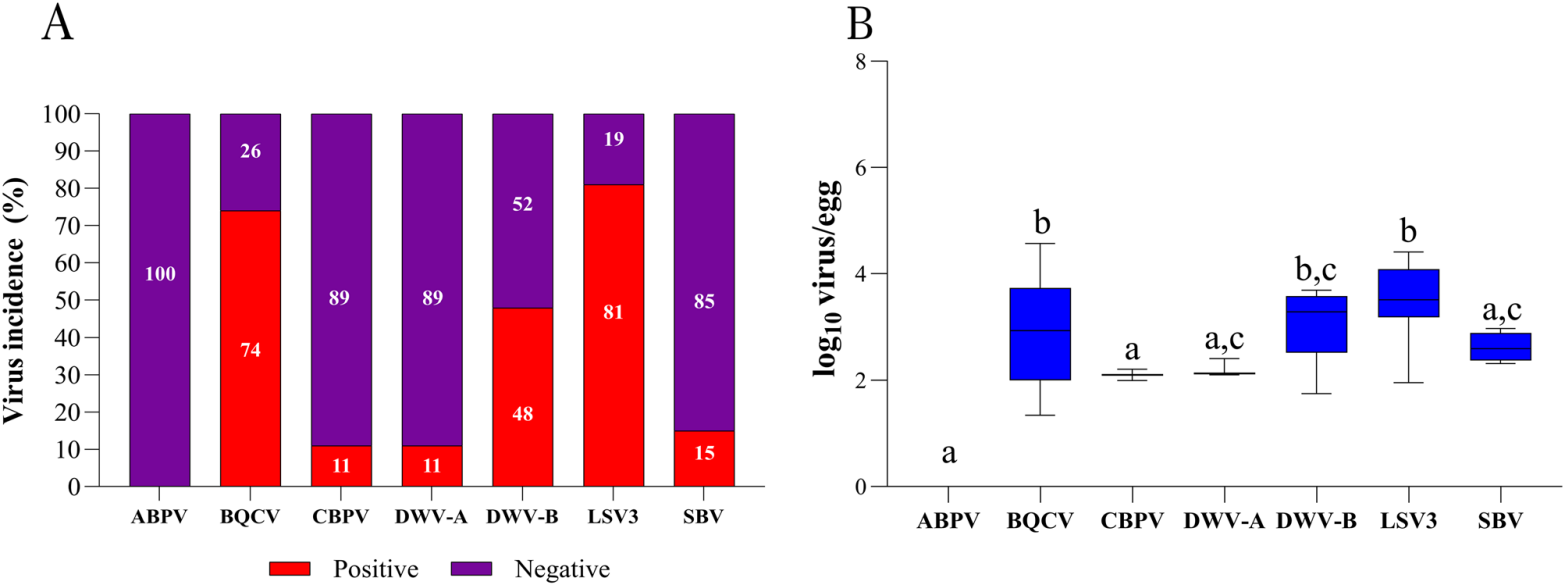
The virus prevalence (**A**) and the viral load (**B**) for ABPV, BQCV, CBPV, DWV-A, DWV-B, LSV3, and SBV in egg samples. Letters denote significant differences between the viruses, determined by the Kruskal–Wallis nonparametric test (statistic = 83.46), followed by Dunn’s multiple comparisons test (p < 0.0001).

Upon individual analysis of eggs, larvae, pupae, and queens, variations in virus incidence and virus load among distinct developmental stages were statistically different (apparent). In eggs, the highest virus incidence was detected for LSV3 (81%), and BQCV (74%). The incidence of other viruses was less than 50% (Fig. 2A). No egg samples tested positive for ABPV-positive. Concerning the virus load (mean of log_10_ virus/egg), a strong statistically significant difference (p < 0.0001) was observed for BQCV (2.86 log_10_ copies/egg), DWV-B (3.07 log_10_ copies/egg), and LSV3 (3.50 log_10_ copies/egg), compared to other viruses (Fig. 2B). No statistically significant difference was observed between BQCV, DWV-B, and LSV3 (p > 0.09).

**Figure 3:**
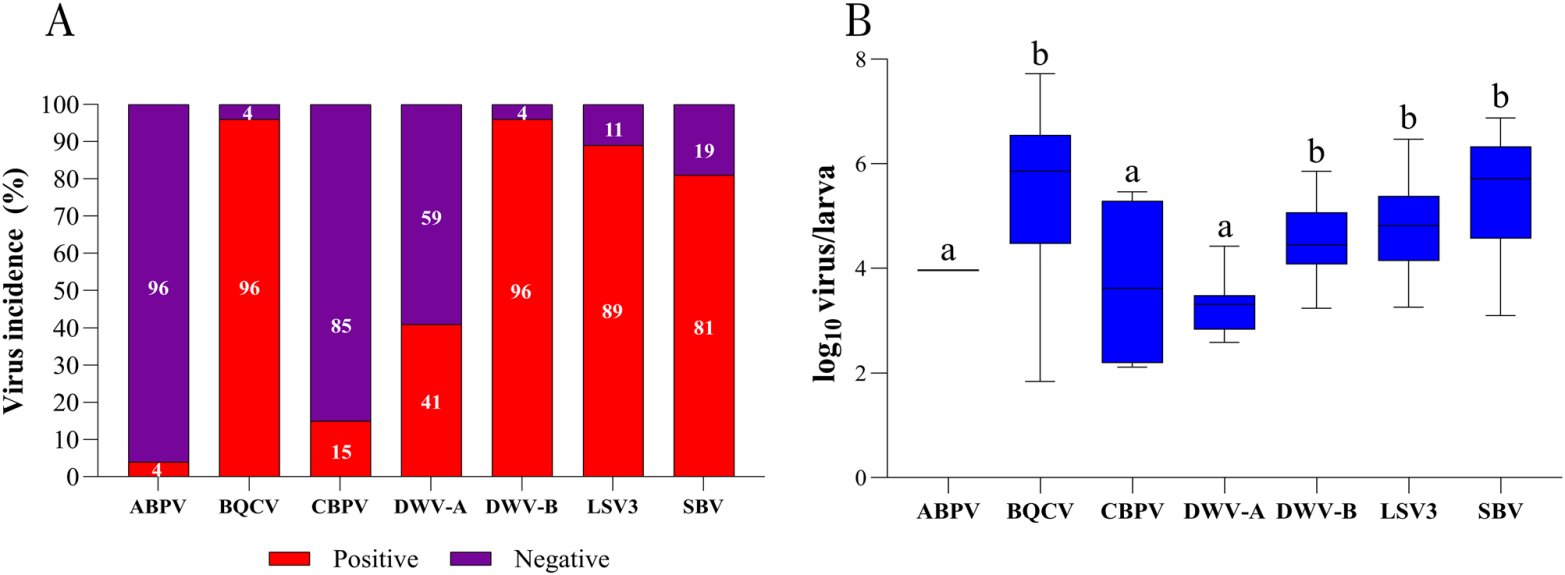
The virus prevalence (**A**) and the viral load (**B**) for ABPV, BQCV, CBPV, DWV-A, DWV-B, LSV3, and SBV in larva samples. Letters denote significant differences between the viruses, determined by the Kruskal–Wallis nonparametric test (statistic = 110.6), followed by Dunn’s multiple comparisons test (p < 0.0001).

Figure 3A summarizes the virus incidence in the larvae samples, where high incidences were observed for BQCV (96%), DWV-B (96%), LSV3 (89%), and SBV (81%). The viral load for BQCV (5.50 log_10_ copies/larva), DWV-B (4.53 log_10_ copies/larva), LSV3 (4.82 log_10_ copies/larva), and SBV (5.47 log_10_ copies/larva) were statistically significantly different (p < 0.0001) when compared to other viruses, as highlighted in Fig. 3B. There was no statistically significant difference between BQCV, DWV-B, LSV3, and SBV (p > 0.9999).

**Figure 4:**
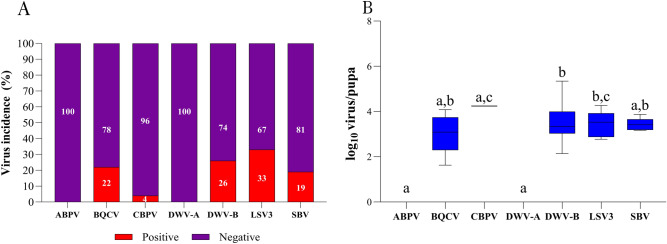
The virus incidence (**A**) and the viral load (**B**) for ABPV, BQCV, CBPV, DWV-A, DWV-B, LSV3, and SBV in pupa samples. Letters denote significant differences between the viruses, determined by the Kruskal–Wallis nonparametric test (statistic = 27.96), followed by Dunn’s multiple comparisons test (p < 0.0001).

In pupae samples, all viruses had an incidence of less than 50%, and no ABPV and DWV-A positive samples were detected (Fig. 4A). Regarding viral load, similar to the virus incidence, a low viral load was observed in the pupae samples, as shown in Fig. 4B. The viral load for BQCV (3.01 log_10_ virus/pupa), DWV-B (3.52 log_10_ virus/pupa), LSV3 (3.45 log_10_ virus/pupa), and SBV (3.42 log_10_ virus/pupa) were similar to each other (p > 0.9999). Only one sample tested positive for CBPV (4.24 log_10_ virus/pupa); however, all analyzed samples tested negative for ABPV and DWV-A.

**Figure 5:**
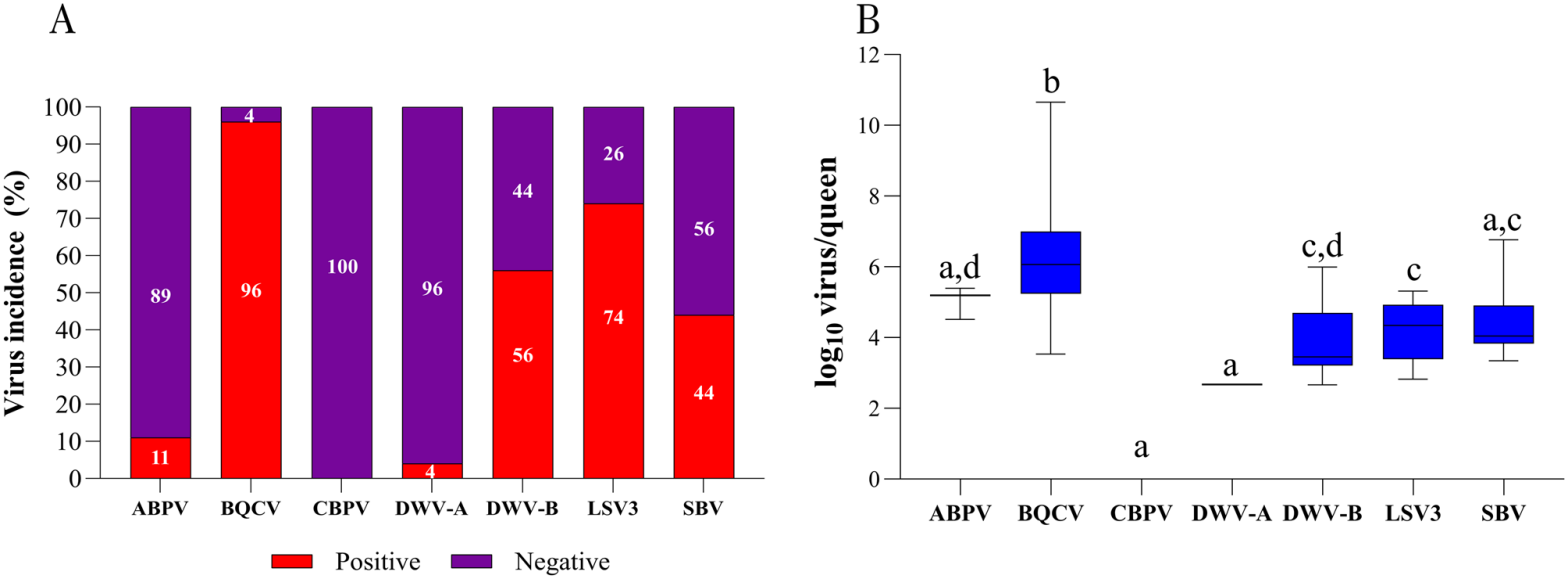
The virus incidence (**A**) and the viral load (**B**) for ABPV, BQCV, CBPV, DWV-A, DWV-B, LSV3, and SBV in queen samples. Letters denote significant differences between the viruses, determined by the Kruskal–Wallis nonparametric test (statistic = 105.3), followed by Dunn’s multiple comparisons test (p < 0.0001).

The samples from the queens exhibited three viruses with an incidence of more than 50% (Fig. 5A). No CBPV-positive sample was found in queens. The highest viral load (6.07 log_10_ copies/queen) was detected in BQCV, which differed statistically from all other viruses (p < 0.0001), Fig. 5B. DWB-B (3.86 log_10_ copies/queen), LSV3 (4.22 log_10_ copies/queen), and SBV (4.44 log_10_ copies/queen) showed no statistically significant differences (p > 0.9999).

### Comparison of virus load

**Figure 6:**
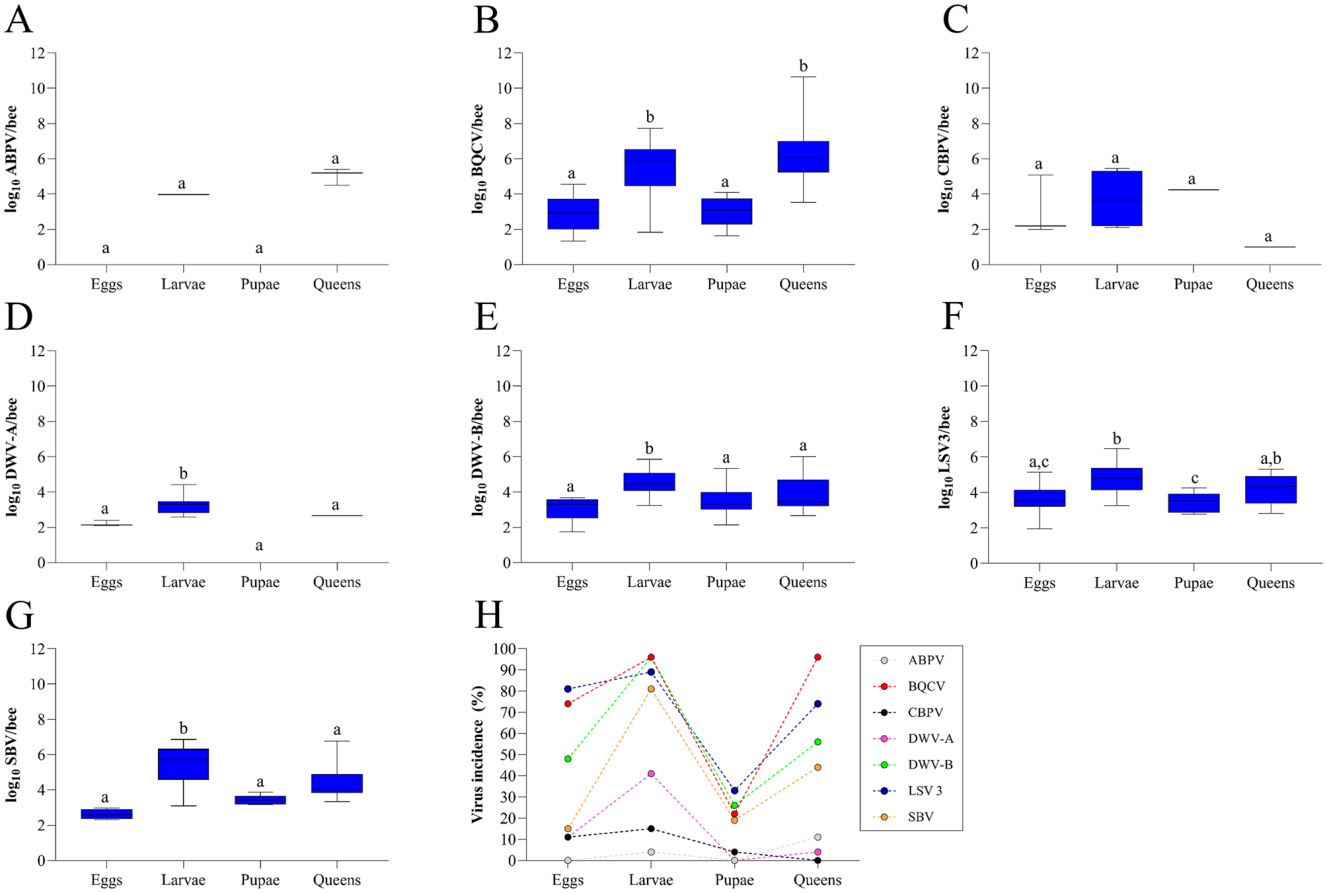
The viral load for ABPV (**A**), BQCV (**B**), CBPV (**C**), DWV-A (**D**), DWV-B (**E**), LSV3 (**F**), and SBV (**G**), as well as the virus incidence (**H**) in different developmental stages for eggs, larvae, pupae, and queens. Letters denote significant differences between the developmental stages, determined by the Kruskal–Wallis nonparametric test, followed by Dunn’s multiple comparisons test (p < 0.0001).

Figure 6 compiles all the data to understand the viral presence at different stages of development. It is worth noting that ABPV (p = 0.0985) and CBPV (p = 0.3316) showed no statistically significant difference across all sampled stages, as depicted in Fig. 6A, C, respectively. BQCV showed the highest viral load in larvae and queens (p < 0.0001) compared to eggs and pupae (Fig. 6B). However, there was no significant difference between larvae and queens (p > 0.9999), and similarly, eggs and pupae exhibited similarity (p = 0.3982). Regarding DWV-A, Fig. 6D highlights that the viral load was significantly higher only in larvae samples compared to other developmental stages (p < 0.0001), while the viral loads of the other stages were similar to one another (p > 0.9999). DWV-B (Fig. 6E) exhibited a higher viral load in larvae (p < 0.0001) compared to other stages, which showed similarity (p > 0.9999). Concerning LSV3 (Fig. 6F), the highest viral load was observed in larvae which were significantly different from eggs and pupae (p < 0.0001). No difference was observed between larvae and queens (p = 0.1392). Figure 6G shows that the viral load of SBV was significantly higher (p < 0.0001) in larvae compared to eggs, pupae, and queens.

Figure 6H compiles the incidence of all viruses tested at different stages of development. It is noted that the BQCV, DWV-B, LSV3, and SBV viruses were more present in the larval and queen stages. The lowest incidences of viruses were observed in pupae.

## Discussion

The present study demonstrates variability in the incidence and viral loads of the tested virus set across the queen's developmental stages. It is worth pointing out that all seven tested viruses were detected (pooled samples), with the highest incidence observed for BQCV, LSV3, DWV-B, and SBV at 72%, 69%, 60%, and 40%, respectively. ABPV, CBPV, and DWV-A were also detected but at relatively low levels. Furthermore, studies have suggested that these viruses may impact the health and productivity of queens^7,31^. Regarding this issue, viruses can be transmitted vertically from queen mother through eggs to the offspring^37^.

Marked by the transfer of viruses from mother to offspring through the egg, such as DWV, vertical transmission can occur either on the egg's surface (transovum) or within the egg (transovarian)^4,31^, which also influences virus virulence^41,42^. Vertical transmission plays a crucial role in facilitating long-term virus persistence, with the outcome being determined by the balance between vertical and horizontal transmission processes^4^. The high incidence of viruses demonstrated in our experiment also appears to be influenced by horizontal virus transmission through common social activities such as visiting flowers, pollen collection, trophallactic interactions, hygienic behavior, grooming, and cannibalism^43–46^. For instance, the parasitic mite *Varroa* serves as a vector for horizontally transmitting DWV to larvae and pupae, which might cause the colony to become weaker and more susceptible to disease^47^. It remains unclear which transmission route prevails in the context of queen-rearing. Further studies are needed to establish vertical versus horizontal virus transmission impacts and modes under specific commercial queen-rearing conditions.

The viral load corresponds to the uptake and manipulation of virus-contaminated food and can increase during repeated ingestion by larvae or adult bees, and also because of the prevalence of varroa infestations^48^. DWV is a well-studied virus found in eggs and larval stages, and positive results are generally linked to varroa infestation^4^. In addition, Žvokelj et al.^35^ demonstrated the presence of ABPV, BQCV, and DWV in worker bees from breeder colonies, nurse colonies, and mating nuclei of *A. mellifera carnica*. These viruses were also found in queen larvae and pupae in nurse colonies. A study performed by Chen et al.^49^ showed that DWV can be detected in eggs, larvae, pupae, and adults. Based on our data, the presence of six viruses was detected in the examined eggs from five apiaries (Fig. 2). ABPV was not detected, whereas the highest incidence was established for LSV3, BQCV, and DVW-B, with significantly higher viral loads compared to SBV, DWV-A, and CBPV. The appearance and quantity of DWV in eggs are primarily associated with vertical transmission^39,40,50^. It is therefore assumed that highly DWV-infected queens can spread infection to their offspring through vertical transmission. Viruses such as SBV and BQCV were also found in queens and simultaneously in eggs^4,51^.

The presence of viruses in eggs can vary across samples collected from queen mother colonies. We found some egg samples infected with only one virus, but also multiple virus infections were detected in eggs, as observed in the tested honey bee colonies in the Belgian honey bee breeding program^52^. In this regard, screening for virus infection in eggs emerges as a good indicator of resilience to virus infection in the colony, supporting the data from the literature^39,40^. Additionally, the SOV trait described by de Graaf et al.^38^ can reflect the degree of vertical transmission at the collection time and appears to be a valuable tool in breeding programs to increase virus resistance.

Testing the same queen mother colonies in our experiment revealed the presence of ABPV in 4% of the larvae. Along with CBPV and DWV-A, the virus loads were lower than those of BQCV, DWV-B, LSV3, and SBV. We observed a high incidence and virus load of SBV in larvae, which could be associated with larval death. This phenomenon is usually not visible within the colony. Sacbrood disease is identified by brood death, with ecdysial fluid found under the larvae's tegument containing significant viral quantities (up to a maximum of 13.35 SBV genome copies/larvae)^53^. BQCV was found in our experiment in 96% of tested live queen larvae, with a viral load of approximately 6 log_10_ genome copies. In other experiments, 8 log_10_ BQCV genome copies in brood induced 67% queen larvae mortality^53^.

Infection with ABPV affects the emergence of infected larvae and pupae and also impacts adult nurse bees, leading to excessive loss of adult bees. Specifically, ABPV aggregates in the hypopharyngeal glands^54^. ABPV was absent again in pupae, as is the same finding with DWV-A. Generally, the virus incidence was lower in pupae compared to larvae or emerged queens. The lowest threshold for ABPV loads was set at 5 log_10_ genome copies/bee. This result is indicative that honey bees have a low tolerance to ABPV replication. A relatively low incidence of ABPV was detected in our brood or queen samples, with approximately 4 log_10_ genome copies/larvae on average and 5 log_10_ genome copies/young queen. Our results obtained in real queen rearing conditions showed lower (in larvae) or similar (in queens) threshold levels compared to levels found by the European Union Reference Laboratory for honeybee health^53^.

We observed the highest percentage (33%) of pupae that tested positive for LSV3. Studies performed in Belgium^55^, Spain^56^, and the USA^57^ have shown LSVs are spread worldwide and, in some cases, associated with poor colony health and reduced populations of native and wild bees in some geographic locations^58^. Previous studies have detected high viral loads of SBV (above 10^9^ copies/head) in dead pupae, indicating a high tolerance to SBV replication^53^. On the other hand, the DWV variant alone cannot induce mortality during the pupal stage^59^. Infected pupae with DWV can develop until emergence^60^. In our study, 26% of the tested pupae were positive for DWV-B, and it appears to dominate DWV-A through a viral competition mechanism, leading to a new viral equilibrium associated with lower virulence^61^.

It has been proven that DWV-B is more virulent than DWV-A and has recently been described as rapidly expanding worldwide^62^. The incidence of DWV-B also indicates increased resistance of colonies to mite infestations, suggesting that the virus recombinants have been adapted to being vectored by the varroa mite^63,64^. It was also established that slightly increased pupal mortality, compared to controls, was caused by both DWV-A and DWV-B inoculation^65^. An interesting and important finding for apiculture is that DWV variants and varroa mites are considered predictive markers of colony collapse and winter losses^66–68^.

In contrast to other pathogens that typically induce clinical manifestations, the viruses did not cause any clinical symptoms in brood development or adult bees by the time of sampling in our experiment^69–71^. Nevertheless, viral infections were identified as a significant threat to apiculture, leading to damage at different developmental stages of honey bees, such as eggs, larvae, pupae, adult workers, drones, or queens^32^. Clinical and even more subclinical alterations during queen rearing are crucial indicators of potential malformations in the reared queens in queen rearing operations. Viral infections are often associated with other bee pathogens, such as *V. destructor* and *Tropilaelaps* mites, as well as *Aethina* *tumida*^44,72^. Viral infections during queen development can result in poor queen quality, leading to smaller colony size, a weakened immune system in worker bees, increased queen supersedure^73,74^, and ultimately, colony mortality^75^.

Furthermore, our results on the presence of viruses in queen's eggs have potential implications for evaluating the quality of reared queens in terms of their viral infections and longevity. These aspects, including the appearance of viruses and their impact on queen quality, need to be further studied in real queen-rearing conditions. Additionally, it is important to highlight that the eggs are an efficient indicator of the virus incidence during queen rearing.

## Materials and methods

### Selection of queen breeder’s apiaries

Five queen breeders of the Carniolan honey bee (*A. mellifera carnica*), with known general management practices, were selected in different locations in Slovenia to participate in the present study. The apiaries were located in Starše (46°27′13.5" N 15°47′45.2" E), Apače (46°22′37.0" N 15°48′09.0" E), Murska Sobota (46°43′44.2" N 16°10′34.8" E), Lukovica (46°07′42.5" N 14°39′31.9" E), and Ptujska Gora (46°20′19.6" N 15°45′00.6" E).

Each apiary location corresponds to a queen breeder, and all colonies were housed in national Slovenian standard AŽ hives (Alberti-Žnideršič). The colonies were treated against varroa during the broodless winter period prior to the study, with an oxalic acid solution applied by trickling. The varroa mites were not seen on adult bees, nor any other symptoms of disease were observed. In the present study, a total of 27 colonies were utilized, with an average of five colonies per queen breeder.

### Material sampling—eggs, larvae, pupae and queens

Throughout the breeding period in 2022, corresponding to the spring and summer in the Northern Hemisphere, a comprehensive range of 108 samples was collected from all previously mentioned locations. Each of the 27 colonies contributed four samples, consisting of eggs, larvae, pupae, and queens (27 colonies × 4 samples each = 108 samples).

The sampling procedure involved direct collection from queen mother colonies using forceps. On each sampling date, a pool of 12 eggs up to three days old were carefully sampled from each individual queen mother honey bee colony. Additionally, a pool of 12 larvae up to five days of development, 12 pupae with eye pigmentation ranging from pink to light brown up to 13 to 15 days of development, and 4 newly emerged queens were sampled from each mother colony and stored for analysis. Queen’s developmental stages (larvae, pupae, queen) were kept in a honey compartment (top part) in the national Slovenian standard AŽ beehive, while mother queens were housed in a brood (bottom) compartment. Each mother queen hive had both compartments divided by a queen excluder. Roughly two thirds of the queen excluder in each hive was covered with a screen to minimize the transmission of queen pheromones from the brood compartment. This practice adheres to standard queen rearing procedures.

To ensure proper preservation, the collected material was stored in sterile plastic bags within a portable refrigerator, subsequently frozen at − 80 °C at the Faculty of Agriculture and Life Sciences (FKBV)—University of Maribor (UM) until further investigation.

### Sample preparation

After defrosting, the samples were carefully separated and processed based on their developmental stage. A total of 12 eggs, 12 larvae, 12 pupae, and 4 newly emerged queens were placed in specific tubes. The eggs were carefully transferred to microtubes containing 1 mL of RPMI 1640 medium (Gibco, Paisley, UK) supplemented with beads (MagNA Lyser Green Beads, Roche). Individual tubes were allocated for each larva, pupa, and queen, containing 5 mL of the same medium (Ika Tube—DT20/50). The tubes were then incubated at room temperature for 30 min and then homogenized using a Minilys^®^ personal homogenizer for eggs and Ultra-Turrax^®^ Tube Drive for larvae, pupae and queens. After that, samples were centrifuged for 15 min at 2500×*g*; 2 mL of supernatant was stored from each sample as a suspension for further viral RNA extraction.

### RNA extraction using the King Fisher technology

The total RNA was extracted from 200 µl of suspension from each sample using the MagMax Core kit with the King Fisher instrument (Thermo Fisher Scientific, ZDA) and diluted in 80 µl of elution buffer, according to the producer's instructions.

### Honey bee viruses’ detection by using specific RT-qPCR’s

Molecular analyses were performed by RT-qPCR methods for ABPV, BQCV, CBPV, DWV-A, DWV-B, LSV3, and SBV detection and quantification^16,53^. The reverse transcription with RT-qPCR assay was performed in a single step using the QuantiNova^®^ Pathogen + IC Kit (Qiagen, Hilden, Germany). The RT-qPCR mix was composed of 5 µl QuantiNova Master Mix, 2 µl 10× Internal Control (IC) Probe Assay, 1 µl IC (1:100), 4.5 µl deionized water, 1 µl forward primer (200 nM), 1 µl reverse primer (200 nM), and 0.5 µl probe (100 nM), and 5 µl of extracted RNA, making a total final volume of 20 µl. Thermal cycling was carried out using an Mx3005P thermocycler (Stratagene, La Jolla, USA) with the following conditions: 20 min at 50 °C, 2 min at 95 °C, followed by 45 cycles of 15 s at 95 °C, 30 s at 60 °C, and 30 s at 60 °C. In every run, a positive control was included, prepared as a mixed suspension of previously determined positive field samples of seven different viruses (ABPV, BQCV, CBPV, DWV-A, DWV-B, LSV3, and SBV). Additionally, a negative control was prepared and used in the same way as a positive. Each negative control consisted of only 200 µl of RPMI 1640 medium (Gibco, Paisley, UK) in aliquots. The internal mRNA controls for honey bees or varroa were not used, as the efficiency of all qPCR protocols had already been demonstrated previously by Šimenc et al.^16^, and the present study followed the same procedure.

Known copy numbers of the standard for each virus, with tenfold dilutions from 10^–3^ to 10^–7^, were prepared and added in each RT-qPCR run. The exact number of RNA viral molecules in individual samples was calculated for positive samples (Cq ˂ 40) from the standard curve for each of seven honey bee viruses.

Results for each sample were analyzed using MxPro—Mx3005P v4.10 software (Stratagene, La Jolla, USA), and the exact copy number was determined. Initially, the results were expressed as the number of detected viral copies in 5 µl of extracted RNA. Subsequently, each result was calculated to log_10_ viral copies per queen/egg/larva/pupa, using the following equation: number of copies/queen/egg/larva/pupa = copies/5 µl extracted RNA × (1(queen)/0.1(egg)/0.5(larva or pupa) for sample preparation) × 5 (RNA extraction) × 16 (qPCR mix) =  × 80 (for queen)/ × 8 (for egg) × 40 (for larva or pupa) = 1.9 log_10_ (queen)/0.9 log_10_ (egg)/1.6 log_10_ (larva or pupa). These conversion factors were determined based on previously published recommendations^53^ and the volumes used in RT-qPCR assays in this study.

### Statistical analysis

The data were analyzed using the GraphPad Prism 10.2.2 (397) software (GraphPad Prism Software, Inc). A significance level of p < 0.05 was considered to indicate a statistically significant difference. All the obtained data were first analyzed by the Shapiro–Wilk normality test. Due to non-normality distributions, the data were analyzed by the Kruskal–Wallis test followed by the Dunn’s post hoc test for multiple comparisons. The data are graphically represented with mean ± standard error.

### Supplementary Information

Supplementary Table S1.

## Data Availability

All requests for data or datasets should be addressed to Caio E. C. Domingues.
