# Supplementary material for: Eggs sampling as an effective tool for identifying the incidence of viruses in honey bees involved in artificial queen rearing
Source: Sci Rep. 2024 Apr 26;14:9612. doi: 10.1038/s41598-024-60135-1 (PMC11053070; doi:10.1038/s41598-024-60135-1)
Supplement: Supplementary file 1 — Supplementary Table S1. [file 41598_2024_60135_MOESM1_ESM.docx]

**SUPPLEMENTARY INFORMATION**

**Eggs sampling as an effective tool for identifying the incidence of viruses in honey bees involved in artificial queen rearing**

Caio E.C. Domingues^1^, Laura Šimenc^2^, Ivan Toplak^2^, Dirk C. de Graaf^3^, Lina De Smet^3^, Wim Verbeke^4^, Luc Peelman^5^, Leticia S. Ansaloni^1^, Aleš Gregorc^1^

^1^ Faculty of Agriculture and Life Sciences, University of Maribor, Pivola 10, 2311 Hoče, Slovenia

^2^ Institute of Microbiology and Parasitology, Veterinary Faculty, University of Ljubljana, Gerbičeva 60, 1000 Ljubljana, Slovenia

^3^ Laboratory of Molecular Entomology and Bee Pathology, Ghent University, Krijgslaan 281 S2, 9000 Ghent, Belgium

^4^ Department of Agricultural Economics, Ghent University, Coupure Links 653, 9000 Ghent, Belgium

^5^ Laboratory of Animal Genetics, Department of Veterinary and Biosciences, Ghent University, Heidestraat 19, 9820 Merelbeke, Belgium

**Supplementary Table S1.** Spearman’s correlation analysis of seven viruses detected in pooled samples (eggs, larvae, pupae, and queens) of Apis mellifera carnica collected from five queen breeders.

|  |  | **ABPV** | **BQCV** | **CBPV** | **DWV-A** | **DWV-B** | **LSV 3** | **SBV** |
| --- | --- | --- | --- | --- | --- | --- | --- | --- |
| **ABPV** | Spearman's rho | — |  |  |  |  |  |  |
|  | df | — |  |  |  |  |  |  |
|  | p-value | — |  |  |  |  |  |  |
| **BQCV** | Spearman's rho | 0.160 | — |  |  |  |  |  |
|  | df | 106 | — |  |  |  |  |  |
|  | p-value | 0.098 | — |  |  |  |  |  |
| **CBPV** | Spearman's rho | -0.059 | **0.232*** | — |  |  |  |  |
|  | df | 106 | 106 | — |  |  |  |  |
|  | p-value | 0.544 | 0.016 | — |  |  |  |  |
| **DWV-A** | Spearman's rho | 0.074 | 0.185 | **0.197*** | — |  |  |  |
|  | df | 106 | 106 | 106 | — |  |  |  |
|  | p-value | 0.446 | 0.056 | 0.041 | — |  |  |  |
| **DWV-B** | Spearman's rho | 0.079 | **0.406***** | 0.144 | **0.291**** | — |  |  |
|  | df | 106 | 106 | 106 | 106 | — |  |  |
|  | p-value | 0.417 | < .001 | 0.136 | 0.002 | — |  |  |
| **LSV 3** | Spearman's rho | 0.040 | **0.705***** | **0.289**** | 0.135 | **0.488***** | — |  |
|  | df | 106 | 106 | 106 | 106 | 106 | — |  |
|  | p-value | 0.682 | < .001 | 0.002 | 0.165 | < .001 | — |  |
| **SBV** | Spearman's rho | **0.205*** | **0.631***** | 0.186 | **0.277**** | **0.484***** | **0.529***** | — |
|  | df | 106 | 106 | 106 | 106 | 106 | 106 | — |
|  | p-value | 0.034 | < .001 | 0.054 | 0.004 | < .001 | < .001 | — |
| Note: ***** p < .05, ****** p < .01, ******* p <.001. | | |  |  |  |  |  |  |
